# 

**Published:** 1890-12

**Authors:** 


### Society Meetings.

The next meeting of the Buffalo Obstetrical Society will be held December 23d. Subject: Puerperal Mania, by Dr. A. H. Briggs.

### BOOKS AND PAMPHLETS RECEIVED.

A Manual of the Practice of Medicine. By Frederick Taylor, M. D., F. R. C. P., Physician to, and Lecturer at Guy's Hospital; Physician to the Evelina Hospital for Sick Children; Examiner in Materia Medica and Pharmaceutical Chemistry at the University of London. With Illustrations. Philadelphia: P. Blakiston, Son & Co., 1012 Walnut street. 1890.

A Manual of Auscultation and Percussion. Embracing the Physical Diagnosis of Diseases of the Lungs and Heart, and of Thoracic Aneurism. By Austin Flint, M. D., LL. D., Professor of the Principles and Practice of Medicine and of Clinical Medicine in the Bellevue Hospital Medical College, etc., etc. Fifth edition, thoroughly revised. By J. C. Wilson, M. D., Lecturer of Physical Diagnosis in the Jefferson Medical College, etc., etc. Illustrated with wood-cuts. Philadelphia: Lea Brothers & Company. 1890.

Diseases of the Eye. By Edward Nettleship, F. R. C. S., Ophthalmic Surgeon to St. Thomas's Hospital; Surgeon to the Royal London (Moorfield's) Ophthalmic Hospital; Late Ophthalmic Surgeon to the Hospital for Sick Children, Great Ormond street. Fourth American, from the fifth English, edition. With a chapter on Examination for Color-Perception, by William Thomson, M. D., Professor of Ophthalmology in the Jefferson Medical College of Philadelphia. Lea Brothers & Company, Philadelphia. 1890.

Bacteriological Technology for Physicians, with seventy-two figures in the text. By Dr. C. J. Salomonson. Authorized translation from the second revised Danish edition. By William Trelease. New York: William Wood & Company. 1890.

A Compend of Human Anatomy, including the Anatomy of the Viscera. By Samuel O. L. Potter, M. A., M. D., Professor of Theory and Practice of Medicine in the Cooper Medical College of San Francisco; author of A Hand-book of Materia Medica, Pharmacy and Therapeutics, etc., etc., etc.; late A. A. Surgeon U. S. Army. Fifth edition, revised and enlarged, with 117 wood engravings; also an appendix, containing numerous tables and sixteen lithographic plates of the nerves and arteries. The Quiz Compend Series. Philadelphia: P. Blakiston, Son & Company, No. 1012 Walnut street. 1890.

Text-book of Materia Medica for Nurses. Compiled by Lavinia L. Dock, graduate of Bellevue Training School for Nurses; Superintendent of Grace Memorial House. G. P. Putnan's Sons. The Knickerbocker Press. 1890.

Essentials of the Practice of Pharmacy. Arranged in the form of questions and answers, prepared especially for pharmaceutical students. By Lucius E. Sayre, Ph. G., Professor of Pharmacy and Materia Medica of the School of Pharmacy of the University of Kansas. Philadelphia: W. B. Saunders, 913 Walnut street. 1890.

Progressive Exercises in Practical Chemistry. By Henry Leffmann, M. D., Ph. D., Professor of Chemistry in the Woman's Medical College of Pennsylvania, in the Pennsylvania College of Dental Surgery, and in the Wagner Free Institute of Science; Pathological Chemist to the Jefferson Medical College Hospital, and William Beam, M. A., Demonstrator of Chemistry in the Pennsylvania College of Dental Surgery; Associate of the Society of Public Analysts of Great Britain. Illustrated. Philadelphia: P. Blakiston, Son & Company. 1890.

Essentials of Minor Surgery and Bandaging, with an appendix on Venereal Disease. Arranged in the form of questions and answers. Prepared especially for students of medicine. By Edward Martin, A. M., M. D., Instructor in Operative Surgery, University of Pennsylvania; Surgeon of the Howard Hospital; Assistant Surgeon to the

University, etc. Illustrated. Saunders Question Compends, No. 12. Philadelphia: W. B. Saunders, 913 Walnut street. 1890.

Index-Catalogue of the Library of the Surgeon-General's Office, United States Army. Authors and Subjects. Volume XI. Phædronus-Regent. Washington: Government Printing Office, 1890,

The Latin Grammar of Pharmacy and Medicine. By D. H. Robinson, Ph. D., Professor of Latin Language and Literature, University of Kansas. Philadelphia: P. Blakiston, Son & Co., No. 1012 Walnut street. 1890.

Post-Mortems. What to Look for and How to Make Them. By A. H. Newth, M. D., London. Edited, with numerous notes and additions. By F. W. Owen, M. D., Demonstrator of Anatomy in the Detroit College of Medicine. Published by The Illustrated Medical Journal Co., Detroit, Mich.

Cause and Treatment of Sterility in Both Sexes and Fecundation by Artificial Methods. Translated from the French of Dr. J. Gérald, Paris. With notes by Charles Everett Warren, M. D., Boston, Mass. With 200 illustrations designed by Jose Roy. Printed for private use only by the profession; pp. xi.—73.

Saunders' Pocket Medical Lexicon. Being a dictionary of words and terms used in medicine and surgery. Collected from the highest authorities and brought up to present date. By John M. Keating, M, D. (University of Pennsylvania), and Henry Hamilton, author of A New Translation of Virgil's Æneid into English rhyme, etc. Philadelphia: W. B. Saunders, 913 Walnut street. 1890.

Transactions of the Medical and Chirurgical Faculty of the State of Maryland, at its semi-annual session, held at Hagerstown, Md., November, 1889. Ninety-second annual session held at Baltimore, Md., April, 1890.

One Hundred Consecutive Cases of Labor at the Maryland Maternite. With a description of the methods practised in that institution. By George H. Rohé, M. D., Director, and William J. Todd, M. D., Resident Physician. Reprint from Transactions of Medical and Chirurgical Faculty of the State of Maryland. 1890.

A Medico-Legal Case. The People vs. William Manly. By Dr. J. B. Andrews. Buffalo, N. Y. From *American Journal of Insanity*, October, 1890. Utica, N. Y.

The Rotary Element in Lateral Curvature of the Spine. By A. B. Judson, M. D., New York. Reprint from the *Medical Record*, November 1, 1890.

The Effects of Dry Atmosphere on Chronic Inflammation of the Larynx and Nares. By E. Fletcher Ingals, A. M., M. D., Professor of

Laryngology, Rush Medical College, etc., etc. Reprint from *The Journal of the American Medical Association*, October 11, 1890.

Report on the Examination of One Hundred Brains of Feeble-Minded Children. By A. W. Wilmarth, M. D., Elwyn, Pa., Assistant Superintendent of the Pennsylvania Institution for Feeble-Minded Children. Reprint from *The Alienist and Neurologist*, October, 1890.

President's Address: Consideration and Determination of Some of the Conditions Requiring Prompt Opening of the Peritoneal Cavity. By E. E. Montgomery, B. S., M. D., Philadelphia, Pa. Reprint from Transactions of the American Association of Obstetricians and Gynecologists. 1890.

Malaria and the Causation of Intermittent Fever. By Henry B. Baker, M. D., of Lansing, Mich. Reprint from *The Journal of the American Medical Association*, October 18, 1890.

Cornell University College of Agriculture. Bulletin of the Agricultural Experiment Station, Agricultural Division, XXI., October, Toma toes; and XXII. November, on the Effect of a Grain Ration for Cows at Pasture. 1890.

Reciprocity Treaties with Latin-America. Message of the President of the United States and Letter of the Secretary of State submitting the recommendations of the International American Conference. Washington: Government Printing Office. 1890.

International American Conference. Report and Recommendations on Postal and Cable Communication with Central and South America. Washington: Government Printing Office. 1890.

International American Conference. Report and Recommendations Concerning a Plan of Arbitration for the Settlement of Disputes between the American Republics. Washington: Government Printing Office. 1890.

International American Conference. Report and Recommendations Concerning Sanitary and Quarantine Regulations in Commerce with the American Republics. Washington: Government Printing Office. 1890.

Stoddart Bros.' Price List of Surgical Instruments and Physicians' Supplies. Buffalo, N. Y. 1890.

A New Test for Determining the Absence or Presence of Equilibrium of the External Ocular Muscles. By Alvin A. Hubbell, M. D., Buffalo, N. Y. Reprint from the *Buffalo Medical and Surgical Journal*, August, 1890.

HEALTH BULLETINS.

Abstracts of Proceedings of the Michigan State Board of Health. Meeting October 4, 1890.

Newport, R. I. Reports of Deaths and Contagious Diseases for the month of October, 1890.

Abstracts of Sanitary Reports, U. S. Marine Hospital Bureau, Washington, D. C. Volume V., Nos. 44, 45, 46, and 47.

Health in Michigan, October, 1890.

COLLEGE ANNOUNCEMENTS.

Medical College Announcements for 1890-1891 :

Woman's Medical College of Cincinnati.
London Post Graduate School.
Kentucky School of Medicine.

### NOTICE TO CONTRIBUTORS.

We are glad to receive contributions from every one who knows anything of interest to the profession. Articles designed for publication in the Journal should be handed in before the first day of the month.

The Editors are not responsible for the views or opinions of contributors.

All communications should be addressed to

284 Franklin Street, Buffalo, N. Y.

